# Potential Health Risk to Brazilian Infants by Polybrominated Diphenyl Ethers Exposure via Breast Milk Intake

**DOI:** 10.3390/ijerph191711138

**Published:** 2022-09-05

**Authors:** Marília Cristina Oliveira Souza, Paula Pícoli Devóz, João Paulo Bianchi Ximenez, Mariana Zuccherato Bocato, Bruno Alves Rocha, Fernando Barbosa

**Affiliations:** Analytical and System Toxicology Laboratory, Department of Clinical Analyses, Toxicology and Food Sciences, School of Pharmaceutical Sciences of Ribeirao Preto, University of Sao Paulo, Avenida do Cafe s/no, Ribeirao Preto 14040-903, Brazil

**Keywords:** polybrominated diphenyl ether, lactating women, infant exposure, Brazilian breast milk, risk assessment

## Abstract

Polybrominated diphenyl ethers (PBDEs) are ubiquitous flame retardants and are environmentally persistent. PBDEs show endocrine disruption, neurotoxicity, and lower birth weight in infants, and their human body burden has become a public health concern. The infants’ exposure begins in the prenatal period and continues via breast milk ingestion, although, little is known about the factors that may influence this exposure. In this study, PBDE levels in Brazilian breast milk were assessed in 200 lactating women. The risk assessment of infants’ exposure to PBDE was performed through the estimated daily intake (EDI) calculation. The geometric mean (GM) of ∑_PBDEs_ levels was 2.33 (0.14–6.05) ng/g wet weight. At least one PBDE congener was detected in the samples, and the 2,2′,4,4′-tetrabromodiphenyl ether (BDE-47) showed a 100% of detection rate (GM of 1.05 ng/g). Location of residence, maternal level education, monthly salary, and race were positively associated with PBDE levels (*p* < 0.05). The EDI of BDE-47 was higher in Belo Horizonte (8.29 ng/kg/day) than in Viçosa (6.36 ng/kg/day), as well as for the ∑_PBDEs_ (19.77 versus 12.78 ng/kg/day) (*p* < 0.05). Taking the high detection rate of PBDEs in breast milk and their toxicity, continuous studies on infant exposure, fetal growth, and child neurodevelopment are requested.

## 1. Introduction

Polybrominated diphenyl ethers (PBDEs) are environmental pollutants that belong to the group of most-used brominated flame retardant chemicals. These compounds reduce the flammability of the materials with high efficiency and low cost. These synthetic compounds are ubiquitous and found in the human body and environmental samples for many years since they are not chemically bound to the products, and therefore, can migrate into the air, soil, and dust. Regardless of their local use, PBDEs are spread throughout the world [1,2,3,4,5,6]. Owing to the persistence and environmental bioaccumulation of PBDEs, epidemiological studies and concerns by regulatory agencies about the occurrence of potential adverse effects on human health have increased [1,2,7,8]. 

Previous studies have described the potential human health risks after PBDEs exposure, including endocrine disruption, mainly in the thyroid hormones (THs), disruption of calcium signaling, and neurodevelopmental toxicity. Besides, alterations in outcomes in pregnant women such as preeclampsia, lower birth weight in newborns, hepatic disorders, induction of oxidative stress with consequent DNA damage, apoptotic cell death, and carcinogenesis also be observed [1,2,5,6,9,10,11,12,13,14,15]. The hydroxylated metabolites of PBDEs, including 4′-hydroxy-2,2′,4,5′-tetrabromodiphenyl ether (4-OH-BDE-99), and 3-hydroxy-2,2′,4,4′-tetrabromodiphenyl ether (3-OH-BDE-47), showed structural similarity to THs and may change these receptor activities and compete for binding to the thyroid transport proteins [11,16,17,18,19,20].

In non-occupational cases, human exposure to PBDEs occurs via a combination of ingestion, inhalation, and dermal contact. Exposure to PBDEs by diet occurs mainly through the ingestion of dairy products, eggs, fish, seafood, and meat, due to the accumulation of these compounds in fat tissues found in foods of animal origins [1,3,5,6,15,21,22,23,24,25,26]. The infants’ exposure to PBDEs begins in the prenatal period, measured through monitoring of the concentrations in cord blood and the placental tissue, and continues given the presence of PBDEs in breast milk. Infants are a susceptible group of the population to develop neurological problems and their exposure to organic pollutants, such as PBDEs, has become a public health problem. To minimize this exposure, monitoring PBDE levels in breast milk should be a continuous practice worldwide [26,27].

Breast milk is a sample with a high lipid content (3.5% to 4.0%) and has a non-invasive collection method, being considered by the World Health Organization as an ideal matrix for the analysis of the human body burden of the lipophilic compounds, such as PBDEs [15,24,28,29,30]. It is possible to observe an association between high PBDEs concentrations in lipid-rich foods with the PBDEs concentrations determined in breast milk or plasma samples, employing a simple pharmacokinetic model. In addition to the determination of the body burden of PBDEs in lactating women, breast milk also makes it possible to assess the exposure of infants by daily intake. Furthermore, given the implementation of the Stockholm Convention and the consequent environmental release of the persistent pollutants, the PBDE concentrations in human breast milk samples have been reduced in European countries and the USA, updating the PBDE body burden trends [1,7,8,24,26,28,31].

To the best of our knowledge, Brazil has no legislation about the use of these compounds as well as the importation of products containing PBDEs. Besides, the data about infant exposure to these chemicals are scarce. Considering that the biomonitoring data provided by the analysis of breast milk are indicators to evaluate human exposure to PBDEs, this study aimed to determine the background levels of PBDEs in Brazilian breast milk and estimate the health risk by calculating the estimated dietary intake of this compound by infants.

## 2. Materials and Methods

### 2.1. Chemicals/Reagents and Solutions

Seven polybrominated diphenyl ethers congeners were included in this research, 2,4,4′-tribromodiphenyl ether (BDE-28), 2,2′,4,4′-tetrabromodiphenyl ether (BDE-47), 2,2′,4,4′,5-pentabromodiphenyl ether (BDE-99), 2,2′,4,4′,6-pentabromodiphenyl ether (BDE-100), 2,2′,4,4′,5,5′-hexabromodiphenyl ether (BDE-153), 2,2′,4,4′,5,6′-hexabromodiphenyl ether (BDE-154), and 2,2′,3,4,4′,5,6′-heptabromodiphenyl ether (BDE-183). The native standards were purchased from Accustandart^®^ (New Haven, CT, USA). The 4,4′-dibromo diphenyl, from Supelco–Sigma-Aldrich Company^®^ (Bellefonte, PA, USA) was used as an internal standard (ISTD) for PBDEs analyses. All stock solutions were prepared in hexane (for HPLC, ≥99%, Sigma-Aldrich Company^®^, Burlington, MA, USA) and stored at −20 °C until analysis.

### 2.2. Study Population and Samples Collected

The present research configured a multicentric, epidemiological, observational, population-based, and probabilistic sampling study. The samples were collected in the five Brazilian macroregions in the maternal-infant group through cross-sections during the lactation period between 2019 and 2020. For this study, two hundred (*n* = 200) healthy volunteers in the age range of 18 to 42 years old were selected from one Brazilian macroregion (Minas Gerais). Two different centers were included in the analysis, corresponding to two cities with differences in economic and social development, Belo Horizonte (*n* = 100) with a Human Development Index (HDI) of 0.810 and Viçosa (*n* = 100) with an HDI of 0.855. Belo Horizonte is the capital of the state and has 2.72 million inhabitants and is considered the fifth largest productive park in South America. Viçosa has approximately 71 thousand inhabitants, with 92.8% of the population living in urban areas. Both cities have civil construction as the basis of the economy, an important source of exposure to PBDEs for the population. The inclusion criteria for the lactating women’s voluntaries included no previous history of metabolic disorders, renal disease, chronic hypertension, or diabetes mellitus. Lactating women believed to be mentally or physically incapable of participating in this study were excluded.

The collection was realized between 15 and 60 days after childbirth and after the first breastfeeding of the day. During the home visit, the lactating women were instructed on the procedure for collecting human milk samples. The standardized procedure consists of collecting the milk by the nursing mother herself, who will be fasting after washing her hands, to avoid possible sources of contamination, and an amount of 10 mL of milk was collected before the first feeding of the day. It is important to inform the volunteers that the first drops should be discharged before the start of the collection in the previously identified sterile polypropylene bottle. The samples were collected by the research team on a previously scheduled date, separated into 2 mL aliquots, stored at −20 °C in the respective collaborating centers, and immediately sent to Ribeirao Preto, where they were stored at −80 °C until the analysis to preserve their stability for several years.

A questionnaire was applied to the population of this study in a face-to-face interview by trained personnel. Data on demographic and socioeconomic characteristics (age, marital status, years of formal education, occupation), and lifestyles (including alcohol intake and illicit drug use) were included in the questionnaire. The characteristics of the study subjects are described in Table 1. These data were used in the statistical analysis described in Section 2.6 of the materials and methods. This research was approved by the Institutional Ethical Review Board of the School of Pharmaceutical Science of Ribeirao Preto, University of Sao Paulo, Brazil (CAAE 96788518.9.1001.5403). All participant information was kept confidential. The infant’s parents were informed about the study, guaranteeing them the right to participate or not. After the legal guardian agreed to participate, they signed written informed consent.

### 2.3. Sample Preparation and Instrumental Analysis

This biomonitoring study evaluated the concentrations of ∑tri to hepta-BDE in Brazilian breast milk samples. The analytical method was based on reported studies by Souza et al. (2021) [32], with adaptations. Initially, lyophilized breast milk samples were spiked with 20 µL of internal standard and extracted with 2 mL of hexane. The lipid remotion included the first step of matrix clean-up and was performed employing 1 mL of sulfuric acid in ultrasound for 15 min. After this procedure, the organic phase was separated and evaporated and the residue was reconstituted in 500 µL of acetonitrile. After that, the microextraction by packed sorbent (MEPS) was carried out with a C18 cartridge as solid sorbent material (SGE–Analytical Science^®^, Ringwood VIC 3134, Australia) coupled to a syringe of a volume of 50 µL, realizing an additional clean-up and preconcentration of the PBDEs. Acetonitrile was used to condition the C18 column. All sample volume was loaded in the cartridge and eluted with hexane for the GC-MS analysis. To avoid the carryover effect the elution was realized in duplicate and the column was washed with acetone and hexane.

The instrumental analysis of PBDEs was performed employing gas chromatography-mass spectrometry (GC-MS) by Thermo Fisher Scientific^®^ (Waltham, MA, USA). The chromatographic separation was carried out on a GC analytical column FS-CAP SLB-5MS (30 m × 0.25 mm × 0.25 µm) from Sigma-Aldrich^®^, and helium was used as carrier gas. The detection system was mass spectrometric–ISQ single quadrupole, operated in electron impact ionization mode (EI). The data were acquired using Full Scan mode with *m*/*z* of 100 to 800, and the transitions Selective Ion Monitoring (SIM) of each analyte and internal standard (4,4′-dibromo diphenyl) as mentioned in Souza et al. (2021) [32].

### 2.4. Quality Assurance and Quality Control

To evaluate the accuracy and the method performance, quality control procedures were included in these analyses. Two quality control samples were realized in the PBDEs determination for every 25 samples analyzed. These quality control samples were prepared in the laboratory using low and higher concentrations (0.6 ng/g wet weight and 96 ng/g wet weight). The results of coefficients of variation obtained in these quality control samples were lower than 15%. To measure the occurrence of carryover effects, a vial with hexane was used as a procedural blank, and the results showed values less than the limit of detection in all samples and for all congeners. Quantification of PBDEs was carried out employing the internal standard method. Calibration curves were performed by least-squares linear regression analysis of weighted analysis of the ratio between peak areas of the analytes and the internal standard with eight increasing concentrations in triplicate. Further, a calibration curve was added to every 25 samples analyzed. The range of the calibration curve was 0.2 to 120 ng/g wet weight (ww), with the correlation coefficient (*r*) greater than 0.99 for all PBDE congeners.

The recovery test was performed for all congeners of PBDEs, in triplicate, and concentrations relative to the quality control of low and higher concentrations. The average recovery of PBDEs congeners was 95.7% (92.1%–102.8%). The lower limit of detection (LOD) was calculated and separated for each PBDE congener and based on the three times the signal-to-noise ratio. In this study, the LOD selected was the higher value calculated for the congeners, corresponding to 0.066 ng/g ww. The repeatability and reproducibility of this method were evaluated through intra- and inter-assay, employing six extracted samples in the same analytical run and three different runs on different days, respectively. The coefficient of variation and the values of the percentage of imprecision were less than 15%, showing a methodology with robustness and precision, and allowing a satisfying quantitative analysis of the samples.

### 2.5. Estimated Daily Intake of PBDEs by Infants

The determination of the estimated dietary intake of PBDEs by infants via breast milk was based on a reported study by Cui et al. (2012) [33] and Zhao and Shi (2021) [34]. The evaluation of infant exposure to PBDEs was performed considering only breast milk intake since the infants were aged up to 6 months and fed exclusively via breast milk. The EDIs were calculated by the following equation:

$$\mathrm{Estimated}\;\mathrm{Daily}\;\mathrm{Intake}\;\left(\mathrm{EDI}\right)=\frac{\mathrm{PBDEs}\;\mathrm{concentration}\;\text{×}\;\mathrm{Daily}\;\mathrm{milk}\;\mathrm{consumption}}{\mathrm{Infant}\;\mathrm{body}\;\mathrm{weight}}$$

### 2.6. Data Analysis

Descriptive statistics were used to assess the PBDEs levels in breast milk from Brazilians. The values of the median, geometric mean (GM), and percentiles of PBDEs concentrations are demonstrated as nanograms per gram of milk. The PBDEs concentrations below the LOD were replaced by a value equal to the LOD divided by the square root of 2 [35]. Mann–Whitney U test, Kruskal–Wallis test, and Spearman’s correlation test were chosen for data analysis. The covariates and the study subjects that have been included in descriptive statistics were maternal age, occupation or work, maternal education level, household monthly salary, residence location (urban and rural), and habit of smoking and drinking. All statistical inferences were performed by Statistical Package for Social Science (SPSS) version 20.0 (IBM, Chicago, IL, USA), and statistical tests were considered significant if the two-tailed *p* < 0.05.

## 3. Results and Discussion

### 3.1. Occurrence of PBDE Congeners in Brazilian Breast Milk

To the best of our knowledge, this is the first study to evaluate infant exposure to PBDEs through the determination of these compounds in Brazilian breast milk samples. The concentrations of the PBDEs congeners (ng/g wet weight) are summarized in Table 2. The geometric mean (GM) of ∑_7PBDEs_ in breast milk samples was 2.33 (0.14–6.5) ng/g ww. In all samples, at least one PBDE congener was detected, and the BDE-47 showed a 100% of detection rate with a GM of 1.05 ng/g ww (0.14–3.02 ng/g ww). BDE-47 is present at significant levels in the atmosphere, both in developed and remote areas, making global contamination a real possibility. Most PBDEs have similar properties, with exception of BDE-209, and due to their lipophilic and persistent characteristics, these compounds have a slow elimination in the body. Furthermore, during metabolism and in the environment, through a process of reductive debromination, congeners of higher molecular weight, such as BDE-99 and BDE-100, can form BDE-47. Similarly, BDE-154 can generate BDE-99 and BDE-100, and BDE-153 can form BDE-99, and consequently also may form the BDE-47 [25,32,36,37,38]. Among the other congeners analyzed, the BDE-28, BDE-99, and BDE-183 also showed higher detection rates, corresponding to 47%, 43%, and 35%, respectively. The BDE-153 was the lowest (12%).

The coefficients of Spearman’s correlation are summarized in Table 3. This statistical analysis showed a low positive association between the congeners BDE-28 and BDE-47 (0.281, *p* = 0.006) and a strong BDE-153 and BDE-154 (0.640, *p* = 0.018). These positive associations can be explained by the similarity in the ADME (absorption-distribution-metabolism-excretion) of these two hexa-BDE congeners. On the other hand, the data show a negative correlation between lower and higher brominated BDEs, including BDE-28 and BDE-183 (−0.481, *p* = 0.007) and BDE-47 and BDE-153 (−0.441, *p* = 0.031). This result may indicate a difference in exposure sources and also be due to the processes of reductive debromination during metabolism and in the environment, as mentioned above. However, in Brazil, little is known about the use of PBDEs, as well as about their environmental levels.

Overall, human exposure to PBDEs varies throughout the world and the environmental distribution patterns of their respective congeners depend on the use, production, and current legislation in the different regions of the globe. The individual genetic characteristics may influence the toxicokinetic processes of the chemicals, including absorption, distribution, metabolism, and excretion. Therefore, the variation between the levels of congeners in breast milk might be associated with these differences in the kinetic rates. Besides that, to evaluate de infant exposure worldwide, different PBDE congeners patterns in human breast milk should be taken into account [24,26,31,39,40,41,42]. Although the PBDEs show a wide world distribution in the environment, little is known about the factors that may influence the levels of human exposure.

Considering the data from both cities, the present study showed statistically significant variation (*p* < 0.05) in the concentration of the sum of PBDEs in breast milk from Viçosa (1.56 ng/g) and Belo Horizonte (2.74 ng/g), and for the BDE-47 (0.97 ng/g versus 1.18 ng/g), according to the data given in Table 2. The reason may be related to differential exposure such as dietary differences and differences in indoor and outdoor environments, which were not evaluated in this study. This statistical analysis is summarized in Table 3. The PBDEs levels in the air were higher in urban areas than in rural locations, due mainly to endogenic pollution [1,11]. This study corroborates with the finds in the literature and showed higher concentrations of BDE congeners in urban areas than in rural areas, with statistically significant differences for BDE-28 (*p* = 0.043) and BDE-47 (*p* = 0.022).

The statistical analysis also was performed to evaluate the influence of maternal education and family income on the PBDE levels. The results indicated higher concentrations of BDE-47 (*p* = 0.037) and BDE-154 (*p* = 0.004) in volunteers with high education levels (complete high school) when compared with those with incomplete high school. In addition, BDE-28 showed a positive correlation with family income (0.268, *p* = 0.016). The study reported by Cui et al. (2012) [33] also showed a positive correlation between the higher concentrations of PBDEs determined in Chinese breast milk with high school education levels and family monthly salary. It is important to consider that diet is one the most relevant source of PBDEs exposure for adults, especially food with high lipid content, such as animal-origin food [29,32,39,41,42]. Besides, contact with electronic products and other consumer products containing PBDEs, and dust ingestion may also be considered an important exposure source for the population. Therefore, a population with higher education levels and socioeconomic status presents greater access to consumer goods and balanced nutrition, justifying the higher PBDE concentrations in their organisms. In this study, no significant correlation was found between PBDE levels in Brazilian breast milk and the habit of smoking and drinking, maternal age, and maternal occupation.

Our results (20.9 ng/g lipid for BDE-47 and 46.4 ng/g lipid for ∑_PBDEs_) were compared to previously reported studies in other countries since the current legislation to use and commercialize products containing PBDEs is different according to location, making the exposure patterns variable. For this comparison, a median value (4.1%) of the percentage of lipids in breast milk samples was used to convert the units to ng/g lipid. These values are reported in Table 4. Burtryn et al. (2020) [9] also reported higher values for BDE-47 (25.9 ng/g lipid) and ∑_PBDEs_ (52.9 ng/g lipid) in the United States. In China, Li et al. (2020) [43] determined high levels of ∑_PBDEs_ (39.56 ng/g lipid) in breast milk samples. Even with the ban on the use of PBDEs in the USA and China, the high values can be justified by the high environmental persistence of these compounds. On the other hand, Abdallah and Harrad (2014) [1] and Fromme et al. (2022) [40] showed lower concentrations for both, corresponding to 3.30 ng/g lipid for BDE-47 and 5.95 ng/g lipid for ∑_PBDEs_ in the United Kingdom and 0.31 ng/g lipid for BDE-47 and 5.73 ng/g lipid for ∑_PBDEs_ in Germany, respectively.

### 3.2. Potential Health Risk for Infants on PBDEs Exposure via Breast Milk

Breast milk feeding is essential for the growth, development, and well-being of infants. Breast milk is a major source of PBDEs for infants since these compounds accumulate in maternal adipose tissue and are excreted during breastfeeding. Thus, infants receive a considerable amount of persistent organic pollutants which are stored in the maternal body over decades of exposure. However, the risk assessment of infant exposure to PBDEs and other environmental pollutants considering only breast milk intake may be underestimated, since there are other potential sources of exposure to these pollutants, such as infant formula and dust intake [14,39,40,41,42].

For the determination of daily intake of PBDEs from breast milk, an infant’s daily milk consumption of 700 mL and an average infant weight of 5 kg (first 6 months) were considered. In this study, the EDI was calculated to BDE-47, BDE-99, and ∑_PBDEs_. These values are summarized in Table 5. The EDI values determined in Brazil for ∑_PBDEs_ (12.78 and 19.77 ng/kg/day) were higher than the reported values by Fromme et al. (2022) [40] in Germany (6.7 ng/kg/day). However, these values were smaller than those found in China (49.34 ng/kg/day and 35.3 ng/kg/day) and the United States (176 ng/kg/day) in previous years [31,33,47]. These statements confirm the trend in the reduction in the PBDE levels in the breast milk/human samples, and consequently a decrease in infant exposure over the years. However, it is important to consider other exposure sources to infants, such as dust intake.

To assess neurodevelopment toxicity, the study by Bakker et al. (2008) [48] evaluated the no adverse effect level (NAEL) for the daily intake of BDE-99 by lactational exposure (18.8 to 41.4 ng/kg/day). Our data showed a range of 1.40–10.92 ng/kg/day in Viçosa and 1.61–14.27 ng/kg/day in Belo Horizonte. These values did not exceed 18.8 ng/kg/day, the lower limit of expected human NAEL for neurodevelopmental toxicity. Considering the exposure to BDE-47 (0.98–21.14 ng/kg/day) and to all BDE congeners analyzed (0.98–33.64 ng/kg/day), the values are higher than the lower limit of NAEL. According to Agency for Toxic Substances and Disease Registry (ATSDR), 0.03 mg/kg bw/day is a value of minimal risk level (MRL) for the Penta-BDE formulation, considering oral exposure. Besides, the MRL for lower brominated PBDEs is 0.07 mg/kg bw/day and 10 mg/kg bw/day for deca-BDE-209. However, the Penta-BDE formulation is formed by several congeners and should be considered the sum of all [49]. The EDI values determined in this study were lower than MRL values.

## 4. Conclusions

To the best of our knowledge, this is the first study that provides data about exposure and risk assessment for infants related to PBDEs ingested via breast milk in Brazil. On the other hand, some limitations can be included, such as samples from a single Brazilian state, lack of data about the food consumption of the population, and the use of electronics, which are both important sources of human exposure to PBDEs. At least one PBDE congener was detected in the samples, and BDE-47 showed a 100% of detection rate. The higher levels were determined in Belo Horizonte, a city with greater social and economic development. Taking the environmental persistence, human toxicity, high detection rate, and levels of BDE-47 (GM of 1.05 ng/g ww) in Brazil, more studies on the effects of maternal PBDE exposure on fetal growth and child neurodevelopment are requested.

## Figures and Tables

**Table 1:** Characteristics of the study subjects.

Data	Viçosa (*n* = 100)	Belo Horizonte (*n* = 100)
Age	18–42 years old	18–40 years old
Smoking habit—Yes No	8 92	7 93
Drinking habit—Yes No	8 92	18 82
Place of residence—Urban Rural	94 06	100 0
Education level—Incomplete high school Complete high school	57 43	54 46
Household monthly salary—No income ≤R$ 499 R$ 500–999 R$ 1000–1999 R$ 2000–2999 R$ 3000–3999 R$ 4000–4999 >R$ 5000	7 24 37 16 7 2 3 4	8 16 31 17 13 4 6 5

**Table 2:** Concentrations of PBDE congeners in breast milk samples in both Brazilian locations (Belo Horizonte and Viçosa). The values are expressed as ng/g wet weight.

Breast Milk	BDE-28	BDE-47	BDE-99	BDE-100	BDE-153	BDE-154	BDE-183	∑_7PBDEs_
Viçosa								
Geometric mean	0.62	0.91	0.65	0.58	0.59	0.55	0.76	1.56
Minimum	0.21	0.14	0.20	0.23	0.29	0.25	0.24	1.83
Maximum	2.61	3.02	1.56	1.70	1.21	1.23	1.53	0.14
Median	0.41	0.68	0.63	0.24	0.43	0.45	0.74	4.81
Percentile 25th	0.26	0.36	0.29	0.23	0.31	0.35	0.43	0.88
Percentile 75th	0.78	1.05	0.84	0.31	0.81	0.78	0.98	2.70
Percentile 95th	1.23	2.43	1.26	1.70	1.21	1.15	1.49	4.62
Detection rate—%	41	100	31	09	16	25	24	-
Belo Horizonte								
Geometric mean	1.01	1.18	0.75	0.72	0.40	0.78	0.61	2.74
Minimum	0.23	0.21	0.23	0.21	0.23	0.21	0.20	2.82
Maximum	2.61	2.74	2.11	1.33	0.75	1.85	1.53	0.73
Median	0.86	1.01	0.78	0.72	0.28	0.70	0.56	6.5
Percentile 25th	0.640	0.628	0.470	0.438	0.230	0.440	0.350	1.763
Percentile 75th	1.230	1.655	0.950	0.960	0.525	0.988	0.778	3.730
Percentile 95th	2.25	2.45	1.45	1.33	0.75	1.65	1.12	4.80
Detection rate—%	53	100	55	20	8	30	46	-

**Table 3:** Spearman’s correlation coefficient to assess the interfering factors in the infant exposure to PBDEs via breast milk intake.

Spearman’s Correlation	City ^a^	Age	Residence Location ^b^	Education Level	Family Income	BDE-28	BDE- 47	BDE-99	BDE-100	BDE-153	BDE-154	BDE-183	∑_PBDEs_
City	1	0.034	0.175 *	−0.050	−0.163 *	−0.447 *	−0.245 *	−0.138	−0.268	0.360 *	−0.222	0.189	−0.391 *
Age		1	0.093	0.013	0.033	−0.090	−0.036	−0.055	0.046	0.186	0.086	0.084	−0.088
Residence Location			1	−0.033	−0.98	−0.212 *	−0.163 *	−0.063	−0.245	0.340	0.094	-	−0.164 *
Education Level				1	0.037	−0.054	−0.150 *	−0.149	0.141	0.065	0.396 *	−0.053	−0.089
Family Income					1	0.267 *	0.026	0.106	0.267	0.200	−0.243	−0.123	0.131
BDE-28						1	0.281 *	0.258	0.494	−0.572	0.208	−0.481 *	0.677 *
BDE-47							1	0.002	−0.160	−0.441 *	−0.097	0.204	0.649 *
BDE-99								1	0.364	−0.649	0.011	0.068	0.403 *
BDE-100									1	-	−0.091	0.301	0.482 *
BDE-153										1	0.640 *	−0.522	−0.072
BDE-154											1	0.233	0.346 *
BDE-183												1	0.442 *
∑_PBDEs_													1

* Correlation is significant at the 0.05 level (two-tailed). ^a^: Cities: Belo Horizonte and Viçosa. ^b^: Residence location: urban and rural location.

**Table 4:** Comparison with studies reported in the literature in other countries. The values are expressed as ng/g lipid.

Country	Year of Sample Collection	BDE-47	∑_7PBDEs_	Reference
Brazil	2019–2020	20.9	46.6	This study
USA	2007	25.9	52.9	Burtryn et al. (2020) [9]
China	2006-2007	1.31	11.18	Cui et al. (2012) [33]
China	2011		1.5	Zhang et al. (2017) [6]
China	2014	-	2.87	Chen et al. (2019) [44]
China	2016–2017	0.41	39.56	Li et al. (2020) [43]
China	2011–2018	0.076	1.10	Zhao and Shi (2021) [34]
United Kingdom	2010	3.30	5.95	Abdallah and Harrad (2014) [1]
United Kingdom	2015	-	5.80	Tao et al. 2017 [5]
Ireland	2016–2018	-	1.40	Wemken et al. (2020) [45]
Spain	2012		1.25	Schuhmacher et al. (2013) [46]
Germany	2016	0.31	5.73	Fromme et al. (2022) [40]
Taiwan	2007–2011	0.53	∑_30PBDEs_ = 3.4	Tsai et al. (2021) [14]

**Table 5:** Estimated daily intake of PBDEs by infants. The values are expressed as ng/kg/day.

Breast Milk	∑_7PBDEs_	BDE-47	BDE-99
Viçosa	12.78 (0.98–33.64)	6.36 (0.98–21.14)	4.53 (1.40–10.92)
Belo Horizonte	19.77 (5.11–45.50)	8.29 (1.47–19.18)	5.27 (1.61–14.27)

## Data Availability

All data generated or analyzed during this study are with the corresponding author, and, if necessary, she is available for taking any questions about the datasets and these can be requested by reasonable request.
